# Variant in a Taste Receptor Locus Tied to Changes in the Use of Insomnia Medication

**DOI:** 10.1016/j.bpsgos.2025.100652

**Published:** 2025-11-10

**Authors:** Gudmundur Einarsson, Hannes K. Arnason, Rosa S. Gisladottir, Gyda Bjornsdottir, Thorgeir E. Thorgeirsson, Astros Skuladottir, G. Bragi Walters, Saedis Saevarsdottir, Magnus K. Magnusson, Gisli H. Halldorsson, Audunn S. Snaebjarnarson, Hafsteinn Einarsson, Gardar Sveinbjornsson, Hannes Helgason, Vinicius Tragante, Gudrun A. Jonsdottir, Hildur M. Aegisdottir, Ingileif Jonsdottir, Thorarinn Gislason, Gudmar Thorleifsson, Patrick Sulem, Hreinn Stefansson, Daniel F. Gudbjartsson, Kari Stefansson

**Affiliations:** a Amgen deCODE Genetics, Reykjavik, Iceland; b Department of Mathematical Sciences, Norwegian University of Science and Technology, Trondheim, Norway; c School of Humanities, University of Iceland, Reykjavik, Iceland; d Faculty of Medicine, University of Iceland, Reykjavik, Iceland; e School of Engineering and Natural Sciences, University of Iceland, Reykjavik, Iceland; f Department of Sleep, Landspítali University Hospital, Reykjavik, Iceland

**Keywords:** Electronic health records, Genetics, Personalized medicine, Pharmacogenomics, Z-drugs

## Abstract

**Background:**

Zopiclone and zolpidem are widely prescribed hypnotic medications for insomnia, sharing similar efficacy but differing in side-effect profiles, particularly concerning taste disturbances. Identifying genetic predictors of intolerance to these medications could inform personalized treatment strategies.

**Methods:**

We conducted a genome-wide association study to identify genetic variants associated with switching between zopiclone and zolpidem in 57,669 Icelanders, using electronic prescription data from Iceland (2003–2020), and 6590 British individuals from the UK Biobank (1990–2017). We included individuals who had received at least 3 prescriptions of either drug. We also investigated data on bitter taste perception using quinine taste test data from 2238 Icelandic individuals.

**Results:**

A common sequence variant, rs6488335-G, within the *TAS2R∗* bitter taste receptor gene locus on chromosome 12, was associated with an increased likelihood of switching from zopiclone to zolpidem (Iceland: odds ratio [OR], 1.29; 95% CI, 1.24 to 1.35; United Kingdom: OR, 1.34; 95% CI, 1.12 to 1.59) and a decreased likelihood of switching in the reverse direction. The effect was more pronounced in women (OR_females_, 1.36; 95% CI, 1.29 to 1.44) than in men (OR_males_, 1.19; 95% CI, 1.11 to 1.27). While the variant is associated with bitter taste perception of quinine, conditional analyses suggest that the pharmacogenetic association with drug switching is independent of taste perception.

**Conclusions:**

Our findings indicate that carriers of the rs6488335-G variant, particularly homozygous women, were more likely to switch from zopiclone to zolpidem, potentially due to heightened sensitivity to taste-related side effects. Preemptive genetic testing could guide clinicians in prescribing zolpidem over zopiclone for individuals at risk, thereby reducing health care visits and improving treatment adherence.

## Plain Language Summary

In a genome-wide study of Icelandic and U.K. data, we found a common variant in a taste receptor gene cluster (rs6488335-G) strongly associated with switching from the insomnia drug zopiclone to zolpidem, especially in women, and protective for the reverse switch. Our findings suggest that genetic screening may help personalize hypnotic prescribing, guiding clinicians to avoid zopiclone in genetically predisposed patients, thereby improving adherence and reducing unnecessary health care visits.

Side effects often determine patient adherence to medications. Zopiclone (1) and zolpidem (2) are nonbenzodiazepine drugs that are widely used to treat insomnia and are comparable in terms of efficacy, sleep onset, and latency (3). However, zopiclone is known for its taste-distortion side effect (39.9% of reported side effects compared with 5.8% for zolpidem) (3), which is a common cause of switching between zopiclone and zolpidem. Current U.K. prescription guidelines recommend prescribing whichever medication is less expensive unless the patient experiences any drug-specific side effects (4). Taken together, these observations suggest that switching between zopiclone and zolpidem may reflect a range of factors, including side effects, cost, and availability, with taste-related side effects playing a prominent role.

Taste-related side effects arise from alterations caused by a drug within the chemosensory pathways responsible for taste perception (5); thus, these effects are not the same as experiencing taste from a stimulus but rather a distortion of taste in the absence of a stimulus. Understanding the genetic basis of taste-related side effects could inform personalized prescribing strategies for insomnia. To date, no genetic variant has been found to be associated with drug-related taste distortion. However, previous genetic research on taste perception has implicated an association with bitterness of propylthiouracil in *TAS2R38* on chromosome 7 and bitterness of quinine (6) on chromosome 12. Perception of other bitter-tasting chemicals, such as caffeine, sucrose acetate, and grapefruit, have also been associated with these loci (7,8).

We postulated that genetic sequence variation could influence the likelihood of experiencing taste-related side effects of drugs. Switching between zopiclone and zolpidem, which are both commonly prescribed and comparable in efficacy, cost, and risk profile, may serve as a proxy for drug-related side effects. Such medication-switching phenotypes have previously been applied, for example as proxies for nonresponse to selective serotonin reuptake inhibitors (9,10). We also analyzed taste perception in a secondary analysis to gain insight into the biological mechanisms underlying the observed pharmacogenetic association. Identifying variants that predict side effects could yield information about the underlying molecular biology of drug response and has the potential to guide personalized treatments, thereby resulting in improved patient well-being and lower health care costs.

## Methods and Materials

We curated prescriptions of zopiclone and zolpidem by matching the Anatomical Therapeutic Chemical codes N05CF01 (zopiclone) and N05CF02 (zolpidem) from Icelandic nationwide prescription records (2003–2020) and the UK Biobank (1990–2017). For each individual, we used all available prescriptions recorded in the registry, regardless of whether they represented a single continuous course of treatment or distinct episodes of use. This approach allowed us to capture consistent switching behavior across the full treatment history rather than limiting the analysis to a single treatment episode. We defined an individual as switching from zopiclone to zolpidem if their first recorded prescription was for zopiclone and, after trying zolpidem for the first time, at least 50% of subsequent prescriptions were for zolpidem. This definition was given to try to detect a consistent switch instead of a temporary one, such as a switch caused by a lack of availability of the preferred medication. This is also the reason why we restricted to individuals with at least 3 prescriptions, because it is not possible to infer a consistent switch for 1 or 2 prescriptions. We defined control individuals as individuals whose first medication was zopiclone, but they either never tried zolpidem or <50% of subsequent prescriptions were for zolpidem. We similarly defined a switch from zolpidem to zopiclone. There are multiple ways in which phenotypes are defined for treatment outcomes based on prescription drug data (9), and our focus is on switching because that is indicative of lack of efficacy or side effects.

To explore the biological basis of taste-related side effects, we resorted to previously collected data on bitter taste perception (see Supplemental Methods). We performed a genome-wide association study (GWAS) to identify genetic variants associated with drug switching and bitter taste perception, leveraging logistic regression and linear mixed models.

## Results

A total of 57,669 individuals filled at least 3 prescriptions of either zopiclone or zolpidem. Among these, 42,118 individuals had their first recorded prescription as zopiclone, and we defined 5826 (14%) as switchers to zolpidem (Table 1). Conversely, 15,551 individuals had their first recorded prescription as zolpidem, and 6101 (39%) of those switched to zopiclone. Females comprised 59% of the total study population and were more likely than males to be switchers when the first recorded prescription was for zopiclone (odds ratio [OR], 1.12; 95% CI, 1.06 to 1.19; *p* = 5.7 × 10^−5^).

**Table 1:** Characteristics of the Case and Control Groups in the Switching From Zopiclone to Zolpidem Genome-Wide Association Study

Characteristic	Iceland, deCODE Genetics	United Kingdom, UK Biobank
Cases		
*N*	5826	264
Sex, female	3546 (60.9%)	185 (70.1%)
Birth year	1955 (30)	1949 (14.25)
Age at first recorded prescription, years	52.3 (25.9)	53.7 (13.7)
Controls		
*N*	36,292	6326
Sex, female	21,074 (58.1%)	4117 (65.1%)
Birth year	1945 (25)	1949 (12)
Age at first recorded prescription, years	59.6 (25.1)	56.9 (14.2)

Values are presented as *n*, *n* (%), or median (IQR). Cases were defined as those who switched from zopiclone to zolpidem. Controls were defined as those who consistently used zopiclone.

**Figure 1:**
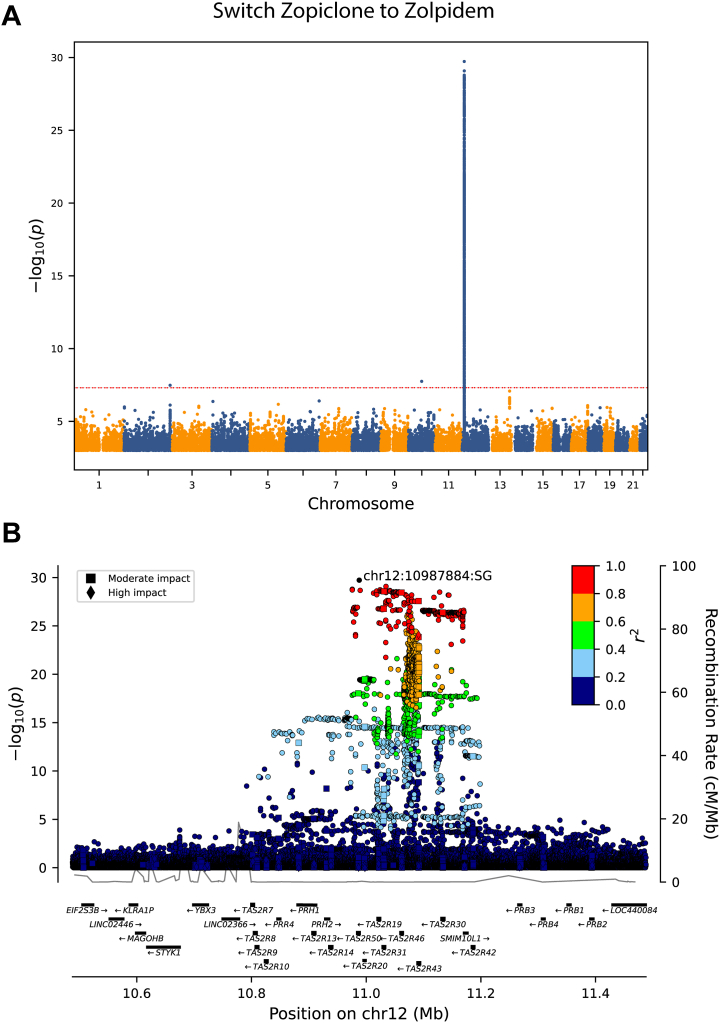
Panel **(A)** depicts a Manhattan plot of the phenotype symbolizing a switch from zopiclone to zolpidem; the red dashed horizontal line indicates a *p* value of 5 × 10^−8^; only the loci on chromosome (chr) 12 is genome-wide significant when we account for multiple testing using a weighted Bonferroni adjustment. Panel **(B)** depicts a locus plot of the region that is associated with the phenotype on chr 12.

**Figure 2:**
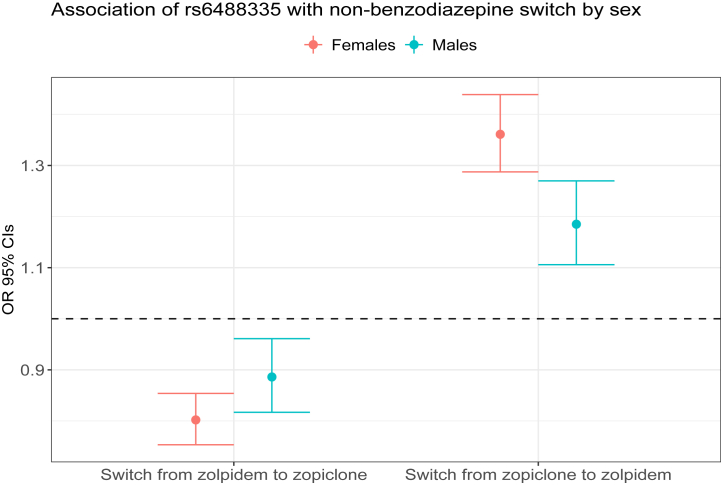
Effects of switching from zopiclone to zolpidem and vice versa stratified by sex. See Table S2. OR, odds ratio.

We identified a genome-wide significant association between the variant rs6488335-G on chromosome 12 (effect allele frequency [EAF]_Iceland_ = 44.6%) and switching from zopiclone to zolpidem (OR, 1.29; 95% CI, 1.24 to 1.35; *p* = 1.9 × 10^−30^) in an additive model (Figure 1). The variant was also associated with a lower probability of switching from zolpidem to zopiclone (OR, 0.83; 95% CI, 0.79 to 0.87; *p* = 6.2 × 10^−13^). The effect of the variant on switching from zopiclone to zolpidem was greater for females than for males (OR_females_, 1.36; 95% CI_females_, 1.29 to 1.44; OR_males_, 1.19; 95% CI_males_, 1.11 to 1.27; *p*_difference_ = 2.2 × 10^−3^) (Figure 2 and Table S2). The region surrounding rs6488335-G contains a cluster of *TAS2R∗* taste receptor genes as well as several *PR∗* genes encoding proline-rich salivary proteins. The variant is noncoding, but it strongly correlated (*r*^2^ > 0.8) with other sequence variants in taste receptor genes, including 6 missense variants: p.Val240Ile, p.Ala227Val, and p.Arg35Trp in *TAS2R31*; p.Leu228Met in *TAS2R46*; p.Leu235Phe in *TAS2R43*; and p.Phe252Leu in *TAS2R30*. The large number of variants associated with switching at the chromosome 12 *TAS2R∗* locus (Figure 1) is consistent with the high linkage disequilibrium (LD) throughout the region, as documented in previous studies (6,8,11) that have linked the locus to the perception of quinine taste. There were no other variants associated with drug switching after we adjusted for rs6488335 in a conditional analysis. We found no other loci associated with the hypnotic drug-switching phenotypes when we controlled for multiple testing using a weighted Bonferroni adjustment (12).

To validate our finding, we defined a drug-switching phenotype in the same way based on drug prescription records of UK Biobank participants (Table 1). Of 7408 individuals who had received at least 3 prescriptions of zopiclone or zolpidem, 6590 started on zopiclone, and 264 (4%) were defined as switchers. rs6488335-G associated with switching from zopiclone to zolpidem (OR, 1.34; 95% CI, 1.12 to 1.59; *p* = 1.2 × 10^−3^), replicating our results from the Icelandic population with a consistent magnitude of effect.

We also performed a GWAS on previously collected data on perceived intensity and pleasantness of the quinine bitter taste among 2238 Icelanders (Figure S2). The lead variant associated with zopiclone to zolpidem switching (rs6488335-G) also associated with less intensity of the quinine taste (effect = −0.25 SD; 95% CI, −0.31 to −0.18; *p* = 1.4 × 10^−14^) and had a nominal association with increased pleasantness ratings of quinine (effect = 0.17 SD; 95% CI, 0.10 to 0.23; *p* = 1.8 × 10^−7^). The strongest association with intensity of the quinine taste at the locus was with the missense variant p.Phe252Leu in *TAS2R30* (effect = −0.27 SD; 95% CI, −0.33 to −0.21; *p* = 1.4 × 10^−17^, rs2599404-A), which is highly correlated with rs6488335-G (*r*^2^ = 0.93) (Figure S2). Sex-stratified analysis of rs6488335 and rs2599404 is included in Table S3. Thus, we confirm in Icelandic data that the drug-switching locus is also related to bitter taste perception. However, the association of p.Phe252Leu with perceived intensity of bitter taste, adjusted for the lead drug-switching variant rs6488335-G was significant (effect_adjusted_ = −0.54 SD; *p* = 3.3 × 10^−5^). Conversely, the association of rs6488335-G with drug switching, adjusted for p.Phe252Leu, was also significant (OR_adjusted_, 1.31; *p* = 1.8 × 10^−3^). This indicates that although the bitter taste perception and drug-switching association signals at the chromosome 12 *TAS2R∗* locus are highly correlated, they represent distinct signals, underscoring a specific link to drug switching at the locus (Table S1). Sex-stratified conditional analyses are provided in Table S4. We found no additional signals for perception of bitter taste at the chromosome 12 *TAS2R∗* locus in conditional analysis.

As taste perception of quinine and propylthiouracil have been described as having high individual variability, we tested whether genome-wide significant variants for quinine and propylthiouracil perception associated with insomnia drug switching. We identified another association with taste perception, represented by the intergenic variant (rs9611875-G, EAF = 4.9%) on chromosome 22, with a large effect on the intensity of quinine taste (effect = −0.52 SD; 95% CI, −0.67 to −0.38; *p* = 1.6 × 10^−12^). The variant was previously associated with decreased liking of bitter ale and beans in a GWAS of food preferences (13). This variant also nominally associated with a lower probability of switching from zopiclone to zolpidem (OR, 0.88; 95% CI, 0.80 to 0.97; *p* = .013).

Next, we tested whether the missense variant p.Ile296Val in the *TAS2R38* gene, which has previously been associated with bitter taste perception of propylthiouracil (rs10246939-C, EAF = 39.6%) (6), also associated with switching from zopiclone to zolpidem, but we found no association (OR, 1.02; 95% CI, 0.97 to 1.06; *p* = .49), indicating that the switch was not mediated by general bitter taste perception.

Given the indication of zopiclone and zolpidem treatment for insomnia, it was relevant to investigate whether there is an association with insomnia at the chromosome 12 *TAS2R∗* gene locus. In a recent meta-analysis of insomnia (14), there was no association with rs6488335-G (*N*_cases_ = 109,548; *N*_controls_ = 277,440; OR, 1.00; *p* = .7), and the closest reported locus on chromosome 12 is more than 5 Mb away from rs6488335.

## Discussion

In this study, we identified a novel pharmacogenetic association, which likely contributes to intolerance of zopiclone due to taste-related side effects. Although not severe, these side effects can contribute to unnecessary discomfort and health care costs. This common variant carries a >30% higher risk of switching from zopiclone to zolpidem and a >80% higher risk for homozygous women, supporting a case for using rs6488335 as a pharmacogenetic marker in prescribing decisions.

The observed sex differences in the effect of rs6488335-G on switching from zopiclone to zolpidem are consistent with reports that taste-distortion side effects are more common and longer lasting in women. Moreover, the sex-stratified analyses indicate that the pharmacogenetic signal for switching is not fully explained by bitter taste perception in women, reinforcing the idea that women may carry additional susceptibility linked to this locus. A double-blind study on taste-disturbing side effects of eszopiclone found that dysgeusia was more intense and longer lasting in women than in men (15). Another study showed that male participants were more willing to endure the side effect due to their improved sleep (16). These findings suggest that women may benefit more from genetic testing when determining insomnia treatment options.

Although high LD complicates pinpointing the causal variant, the consistent association with *TAS2R∗* genes implicates a taste receptor–mediated mechanism. Because we do not have data on drug side effects, the claim that the variant associates with side effects needs to be validated in a future study. Cell-based experiments expressing *TAS2R∗* receptors with the missense substitutions in high LD with rs6488335 could test whether these amino acid changes alter receptor responses to zopiclone, zolpidem, or related bitter compounds. Such work could provide evidence on which genes are involved in the observed pharmacogenetic association. Furthermore, we did not account for concomitant medications other than zopiclone and zolpidem. Therefore, drug interactions or comorbidities could have contributed to switching behavior in some cases. However, the replication of our findings in 2 independent populations suggests that the observed genetic association is robust.

### Conclusions

Our findings highlight the role of common genetic variation in taste receptor genes in shaping treatment choices for insomnia, likely through an increased risk of taste-related side effects. Carriers of the rs6488335-G allele, particularly women who are homozygous, are at substantially increased risk of discontinuing zopiclone. Therefore, in a clinical context, preemptive genotyping could help identify individuals who would benefit from initiating treatment with zolpidem rather than zopiclone. Incorporating preemptive pharmacogenetic testing into clinical decision making could reduce unnecessary health care visits, improve treatment adherence, and enhance patient satisfaction. This study underscores the broader potential of pharmacogenetics in psychiatry and supports the integration of genomic information into routine care. If validated in future studies, testing for rs6488335 may become a useful tool in guiding the selection of hypnotic medications.

## Supplementary Material

Supplemental Methods, Figures S1–S2, and Tables S1–S4

Key Resource Table
