# Supplementary material for: Variant in a Taste Receptor Locus Tied to Changes in the Use of Insomnia Medication
Source: Biol Psychiatry Glob Open Sci. 2025 Nov 10;6(2):100652. doi: 10.1016/j.bpsgos.2025.100652 (PMC12765185; doi:10.1016/j.bpsgos.2025.100652)
Supplement: Supplemental Methods, Figures S1–S2, and Tables S1–S4 [file mmc1.pdf]

SUPPLEMENTARY INFORMATION

Variant in a Taste Receptor Locus Tied to Changes in the Use of Insomnia Medication

Einarsson *et al.*

Supplementary Methods

Study populations:

Iceland: The Icelandic data were collected for several research projects at deCODE genetics. Analysis is restricted to individuals who had reached 18 years of age. Biological samples come from individuals that have provided informed consent for research. The Icelandic Data Protection Authority monitors a sanctioned encryption system that is used to ensure participant confidentiality. The study has been approved by the Icelandic National Bioethics committee (VSN-17-035).

The medication prescription data for zopiclone and zolpidem was acquired from the Icelandic prescription registry maintained by the Directorate of Health, which consists of all electronic medication prescriptions in Iceland since 2003 (VSN-17-035).

The bitter taste data (quinine intensity and pleasantness ratings) were collected in a case-control study on symptoms and physical measures 5 to 18 months after infection of SARS-CoV-2 infection[1] (VSN-15-214 with amendments). We used both cases and controls for the analysis as there was no association between SARS-CoV-2 infection and ratings of quinine intensity and pleasantness. Bitter taste perception was assessed with taste solution (0.005 g quinine hydrochloride in 10 g aqua). Participants were given a plastic spoon with two drops of taste solution and asked to provide an intensity rating of the taste from one to seven (very weak to very strong) and pleasantness rating from one to seven (very unpleasant to very pleasant).

UK Biobank: The UK Biobank (UKB) data contains information on roughly half a million individuals from England, Scotland and Wales. Our analysis included data from individuals of European descent, and their medication prescription records registered by the UK Biobank. All participants in the study willingly gave an informed consent. Access was granted through application number no. 42256.

The medication prescription data was procured from field identifier 42039. A meticulous manual mapping to Anatomical Therapeutic Chemical (ATC) classification codes was conducted, specifically for the codes N05CF01 (zopiclone) and N05CF02 (zolpidem). Using these ATC codes alongside prescription dates, the phenotypes were defined analogously to the method employed for the Icelandic dataset.

Phenotype definition: We defined an individual as switching from using zopiclone to using zolpidem if their first recorded prescription is for zopiclone and, after trying zolpidem for the first time, at least 50% of their collected prescriptions after the switch timepoint are for zolpidem. This definition is given to try to detect a consistent switch instead of a temporary one, such as one caused by a lack of availability of the preferred medication. We defined controls as those individuals whose first medication is zopiclone, but they either never try zolpidem or less than 50% of their prescriptions after first trying zolpidem are for zolpidem. We similarly define a switch from zolpidem to zopiclone.

Sequencing data: In the studies examined, both populations underwent initial whole-genome sequencing to identify genetic variants, followed by imputation into broader datasets using chip-genotyping and long-range phasing[2]. Variant calling was performed with Graphtyper[3]. In Iceland, at deCODE genetics, 64,460 individuals were fully sequenced and 173,025 received chip-genotyping, using technologies such as GAIIx, HiSeq, HiSeqX, and NovaSeq from Illumina. Similarly, the UK Biobank project involved chip-genotyping of all participants, with whole-genome sequencing completed for 131,958 individuals using NovaSeq Illumina systems[4].

Association details: We conducted logistic regression to assess the association of sequence variants with binary phenotypes using an additive genetic model. For the Icelandic cohort, covariates included sex, birth county, current age or age at death (with first- and second-order terms), blood sample availability, and an overlap indicator of individual lifetimes with phenotype

collection periods. In the UK Biobank (UKB) analysis, we adjusted for population stratification with 40 principal components and included age and sex as covariates.

For quantitative traits, association tests employed a linear mixed model via BOLT-LMM, adjusting traits for sex and year of birth and standardizing them for normal distribution. Meta-analyses used a fixed-effects inverse variance method with effect estimates and standard errors to calculate *p*-values for differences in effect sizes across sexes.

To prevent test statistic inflation from cryptic relatedness and population stratification, LD score regression was applied[5]. Additionally, in the GWAS, we controlled for multiple testing using a weighted Bonferroni adjustment, basing weights on the enrichment of functionally impactful variant classes identified in the Icelandic data[6].

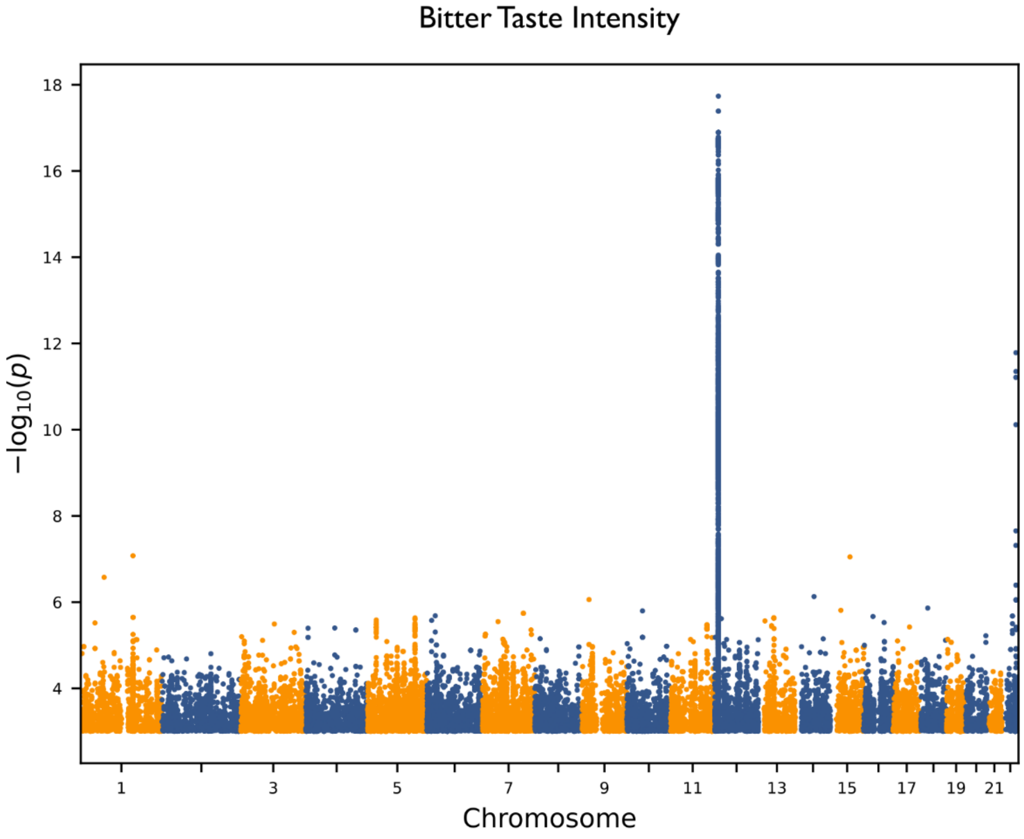

Figure S1 Manhattan plot for the perception of bitter taste phenotype.

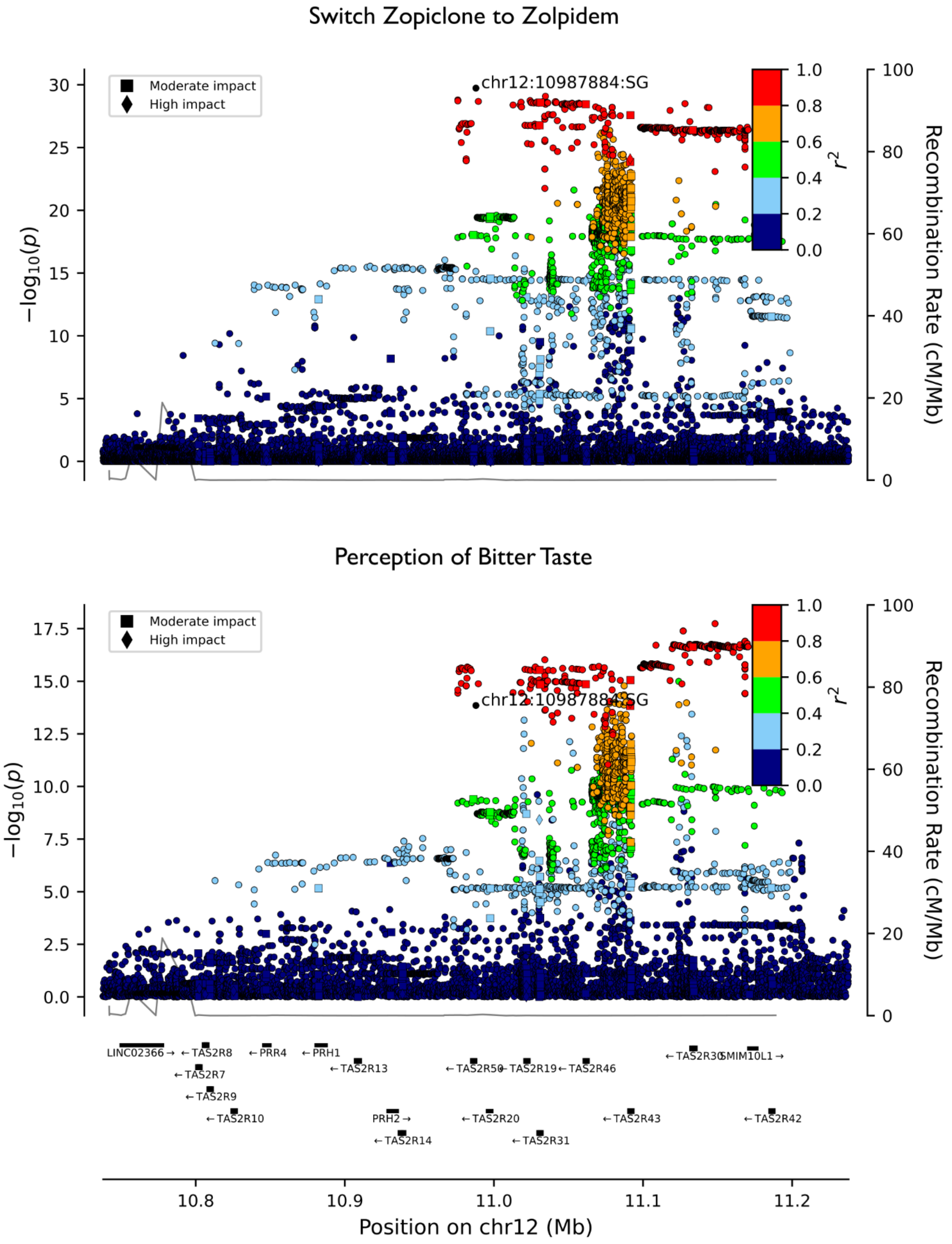

Figure S2 Locus plots of the same region top panel: switch from zopiclone to zolpidem, bottom panel: Perception of bitter taste. Both phenotypes are from Icelandic data.

Phenotype	Chr	Position (hg38)	rsName	EA	OA	Effect	Odds ratio	P-value	Variant covariate
Zopiclone to zolpidem switch	12	10987884	rs6488335	G	T	NA	1.293	1.90E-30	None
Zopiclone to zolpidem switch	12	10987884	rs6488335	G	T	NA	1.312	1.80E-03	rs2599404
Zopiclone to zolpidem switch	12	11133489	rs2599404	A	C	NA	1.273	4.20E-27	None
Zopiclone to zolpidem switch	12	11133489	rs2599404	A	C	NA	0.979	0.80	rs6488335
Intensity of bitter taste	12	10987884	rs6488335	G	T	-0.246	NA	1.40E-14	None
Intensity of bitter taste	12	10987884	rs6488335	G	T	0.283	NA	0.03	rs2599404
Intensity of bitter taste	12	11133489	rs2599404	A	C	-0.270	NA	2.40E-17	None
Intensity of bitter taste	12	11133489	rs2599404	A	C	-0.543	NA	3.30E-05	rs6488335

Table S1 Association of lead switching variant (rs6488335) and lead bitter taste perception variant (rs2599404) with the drug switching and bitter taste perception phenotypes along with conditional analysis.

Phenotype	Chr	Position (hg38)	rsName	Strata	EA	OA	Odds ratio	P-value
Zopiclone to zolpidem switch	12	10987884	rs6488335	All	G	T	1.293	1.90E-30
Zopiclone to zolpidem switch	12	10987884	rs6488335	Males	G	T	1.185	1.50E-06
Zopiclone to zolpidem switch	12	10987884	rs6488335	Females	G	T	1.361	1.40E-27
Zolpidem to zopiclone switch	12	10987884	rs6488335	All	G	T	0.832	6.20E-13
Zolpidem to zopiclone switch	12	10987884	rs6488335	Males	G	T	0.886	3.40E-03
Zolpidem to zopiclone switch	12	10987884	rs6488335	Females	G	T	0.802	5.10E-12

Table S2 Associations of lead drug switching variant (rs6488335) with the drug switching phenotypes stratified by sex.

Phenotype	Chr	Position (hg38)	rsName	Strata	EA	OA	Effect S.D.	P-value
Intensity of bitter taste	12	10987884	rs6488335	All	G	T	-0.246	1.40E-14
Intensity of bitter taste	12	10987884	rs6488335	Males	G	T	-0.318	2.43E-12
Intensity of bitter taste	12	10987884	rs6488335	Females	G	T	-0.180	5.02E-05
Intensity of bitter taste	12	11133489	rs2599404	All	A	C	-0.270	2.40E-17
Intensity of bitter taste	12	11133489	rs2599404	Males	A	C	-0.330	2.81E-13
Intensity of bitter taste	12	11133489	rs2599404	Females	A	C	-0.214	1.30E-06

Table S3 Associations of lead drug switching variant (rs6488335) and lead intensity of bitter taste variant (rs2599404) with the the intensity of bitter taste phenotypes stratified by sex. The unit of the effect is a standard deviation of the intensity of bitter taste phenotype.

Phenotype	Chr	Position (hg38)	rsName	EA	OA	Strata	Odds ratio	P-value	Variant covariate
Zopiclone to zolpidem switch	12	10987884	rs6488335	G	T	Males	1.107	0.48	rs2599404
Zopiclone to zolpidem switch	12	10987884	rs6488335	G	T	Females	1.449	5.37E-04	rs2599404
Zopiclone to zolpidem switch	12	11133489	rs2599404	A	C	Males	1.066	0.65	rs6488335
Zopiclone to zolpidem switch	12	11133489	rs2599404	A	C	Females	0.932	0.51	rs6488335

Table S4 The association of the drug switching phenotype with the lead drug switching variant (rs6488335) conditioned on the lead bitter taste variant (rs2599404), and vice versa, stratified by sex.

References

1. Holm, H. *et al.* Symptoms, physical measures and cognitive tests after SARS-CoV-2 infection in a large population-based case-control study. (2022).
2. Kong, A. *et al.* Detection of sharing by descent, long-range phasing and haplotype imputation. *Nature genetics* **40**, 1068-1075 (2008).
3. Eggertsson, H.P. *et al.* Graphtyper enables population-scale genotyping using pangenome graphs. *Nature genetics* **49**, 1654-1660 (2017).
4. Halldorsson, B.V. *et al.* The sequences of 150,119 genomes in the UK Biobank. *Nature* **607**, 732-740 (2022).
5. Bulik-Sullivan, B.K. *et al.* LD Score regression distinguishes confounding from polygenicity in genome-wide association studies. *Nature genetics* **47**, 291-295 (2015).
6. Sveinbjornsson, G. *et al.* Weighting sequence variants based on their annotation increases power of whole-genome association studies. *Nature genetics* **48**, 314-317 (2016).
